# Counter inhibition between leukotoxins attenuates *Staphylococcus aureus* virulence

**DOI:** 10.1038/ncomms9125

**Published:** 2015-09-02

**Authors:** Pauline Yoong, Victor J. Torres

**Affiliations:** 1 Department of Microbiology, New York University School of Medicine, New York, New York 10016, USA

## Abstract

*Staphylococcus aureus* subverts host defences by producing a collection of virulence factors including bi-component pore-forming leukotoxins. Despite extensive sequence conservation, each leukotoxin has unique properties, including disparate cellular receptors and species specificities. How these toxins collectively influence *S. aureus* pathogenesis is unknown. Here we demonstrate that the leukotoxins LukSF-PV and LukED antagonize each other’s cytolytic activities on leukocytes and erythrocytes by forming inactive hybrid complexes. Remarkably, LukSF-PV inhibition of LukED haemolytic activity on both human and murine erythrocytes prevents the release of nutrients required for *in vitro* bacterial growth. Using *in vivo* murine models of infection, we show that LukSF-PV negatively influences *S. aureus* virulence and colonization by inhibiting LukED. Thus, while *S. aureus* leukotoxins can certainly injure immune cells, the discovery of leukotoxin antagonism suggests that they may also play a role in reducing *S. aureus* virulence and maintaining infection without killing the host.

## Abstract (web summary)

*Staphylococcus aureus* strains produce a family of highly related toxins that puncture the cytoplasmic membrane of susceptible cells. Here, Yoong and Torres show that the toxins can counteract each other in a cell type-dependent manner by forming inactive hybrid complexes, thus modulating *S*. *aureus* virulence.

## Graphical abstract

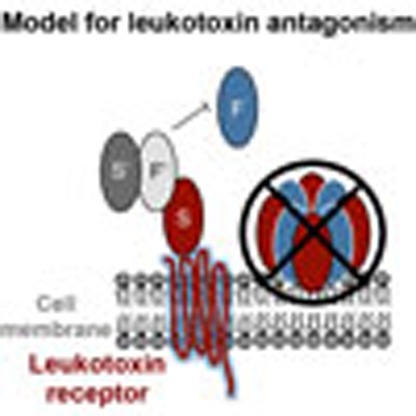

S*taphylococcus aureus* is a bacterial pathogen capable of causing widespread infections and mortality in both the hospital and community settings^1^,^2^. The increased incidence of community-acquired *S. aureus* infection is especially alarming because it occurs in healthy people who lack the underlying risk factors often associated with patients succumbing to *S. aureus* infections in nosocomial settings. The ability for *S. aureus* to invade and colonize multiple sites of infection is attributed to its large arsenal of virulence factors designed to avoid host immune responses, coupled with its rapid acquisition of antibiotic resistance^3^,^4^. Considering the growing numbers of infections caused by methicillin-resistant *S. aureus* (MRSA) strains that now account for over half of all *S. aureus* infections, treatment options are rapidly diminishing^5^. Among the virulence factors encoded by *S. aureus* are a family of five leukotoxins, which include the Panton–Valentine leukocidin (LukSF-PV), leukotoxin ED (LukED), gamma hemolysin (which exists as two toxins: HlgAB and HlgCB) and leukotoxin AB (LukAB, also known as LukGH)^6^. These toxins target an extensive range of the body’s immune cells for destruction, which is thought to play a crucial role in immune evasion by *S. aureus* by promoting bacterial survival and proliferation^6^,^7^.

These leukotoxins are classified as ‘bi-component’ as each is made up of two protein subunits grouped into ‘S’ (for example, LukS-PV, LukE, HlgA, HlgC and LukA) and ‘F’ (for example, LukF-PV, LukD, HlgB and LukB) families, with amino acid sequence conservation within each family ranging from ∼30 to >80% (ref. 6). While these related toxins share many common traits and appear to serve overlapping functions, subtle differences exist between them, indicating distinct roles for each in pathogenesis. First, the leukotoxins differ in their target cell range by recognizing unique cellular receptors, with specificity conferred by the ‘S’ subunit^8^,^9^,^10^,^11^,^12^. The receptor-bound ‘S’ subunit recruits its ‘F’ counterpart, before multimerizing and forming an active β-barrel pore consisting of four ‘S’ and four ‘F’ subunits in the membranes of host cells^13^,^14^, leading to efficient cellular lysis. Cellular receptors for the *S. aureus* leukotoxins have recently been identified. LukED does not exhibit species specificity and similarly targets human and murine cells expressing the G-protein coupled chemokine receptors CCR5, CXCR1 and CXCR2 (refs 9, 10). LukSF-PV specifically recognizes the human chemokine receptors C5aR and C5L2R expressed on poly-morphonuclear neutrophils (PMNs) and monocytes^11^. HlgAB also targets chemokine receptors, but while HlgAB targeting of CCR2 is not species specific, its recognition of CXCR1 and CXCR2 is human specific^15^. As with LukSF-PV, HlgCB also targets C5aR and C5L2R in a human specific manner^15^. LukAB, in contrast to the other leukotoxins, does not target a chemokine receptor, but instead preferentially targets human phagocytes by binding to CD11b, a component of the Mac-1/CR3 integrin^16^,^17^.

LukSF-PV, HlgCB and LukAB preferentially bind to human versions of their receptors, thus making murine cells largely refractory to lysis by these leukotoxins. Adding to this complexity, *S. aureus* clinical isolates also differ in the repertoire of leukotoxins produced. Practically all strains carry genes encoding for the gamma hemolysins and LukAB, while around 85% carry the LukED coding genes^18^,^19^. On the other hand, LukSF-PV coding genes are present only in a small percentage (∼5%) of *S. aureus* strains associated with invasive disease, although they are strongly associated with community-acquired MRSA (CA-MRSA) strains (∼85%) (ref. 20).

The majority of work conducted on *S. aureus* leukotoxins has focused on the contribution of a single leukotoxin at a time. Previous seminal *in vitro* studies have demonstrated that functionally active hybrid leukotoxins can arise from non-standard pairings of purified ‘S’ and ‘F’ type subunits from different leukotoxins^21^,^22^,^23^. The formation of functional hybrid toxins suggest that leukotoxins may influence the activity of one another by synergizing to increase their potency *in vivo* via alteration of receptor specificities. However, how leukotoxins contribute as a group to *S. aureus* pathogenesis *in vivo* is poorly understood.

In this study, we focus on LukSF-PV and LukED, with specific interest in how simultaneous production of these toxins influences the activity of each other. These toxins were chosen because of their extensive sequence identity, despite notable differences in their target cell ranges and species specificities^10^,^11^,^24^,^25^. Here we describe that LukED and LukSF-PV inhibit each other in a cell type-dependent manner via the formation of inactive hybrid toxins that block pore formation by the ‘standard’ toxin. We show that LukED antagonizes LukSF-PV by reducing its cytotoxic potential on human neutrophils, while LukSF-PV inhibits LukED cytotoxicity on murine cells. In addition, LukSF-PV was also found to inhibit LukED haemolysis of both human and murine erythrocytes, thereby preventing the release of nutrients required for *S. aureus* growth. We further demonstrate that deletion of *lukSF-PV* in two genotypes of CA-MRSA, including a strain of the USA300 lineage responsible for the current CA-MRSA outbreak in the United States, increased the virulence and colonization potential of those strains. These phenotypes were found to be dependent on the increased bioavailability and lethal activity of LukED. Altogether, our findings suggest that leukotoxin antagonism may be a strategy by which *S. aureus* regulates its cytotoxic potential to establish long-term infections and perhaps a colonization state.

## Results

### The activity of hybrid leukotoxins is cell-type dependent

The degree of amino acid sequence identity between LukSF-PV and LukED is striking, with the ‘S’ subunits (LukS-PV and LukE) sharing 71% identity and the ‘F’ subunits (LukF-PV and LukD) sharing 82% identity (Supplementary Fig. 1)^7^. Like the standard pairing of LukSF-PV and LukED toxins, the LukS-PV+LukD and LukE+LukF-PV non-standard toxins (hereafter hybrid toxins), have been shown to exhibit cytolytic activity towards human neutrophils and dermonecrosis in rabbits^22^,^23^. Here we also demonstrate the lytic activities of hybrid LukSF-PV and LukED leukotoxins by employing the versatile human promyelocytic HL60 cell line, which can be differentiated into neutrophil-like cells and macrophage-like cells using dimethylsulfoxide and phorbol 12-myristate 13-acetate, respectively^26^ (Table 1 and Supplementary Fig. 2A,B). Our data from undifferentiated and macrophage-like HL60 cells support the notion that the specificity of leukotoxins is conferred by the ‘S’ subunit, which recruits its cognate ‘F’ partner, or the ‘F’ subunit from another leukotoxin, to form functional pores. However, we also observed that the hybrid toxins were unable to lyse PMN-HL60 cells, despite these cells being highly susceptible to lysis by LukSF-PV (Table 1 and Supplementary Fig. 2C).

### LukED inhibits LukSF-PV cytotoxicity towards PMN-HL60 cells

**Figure 1:**
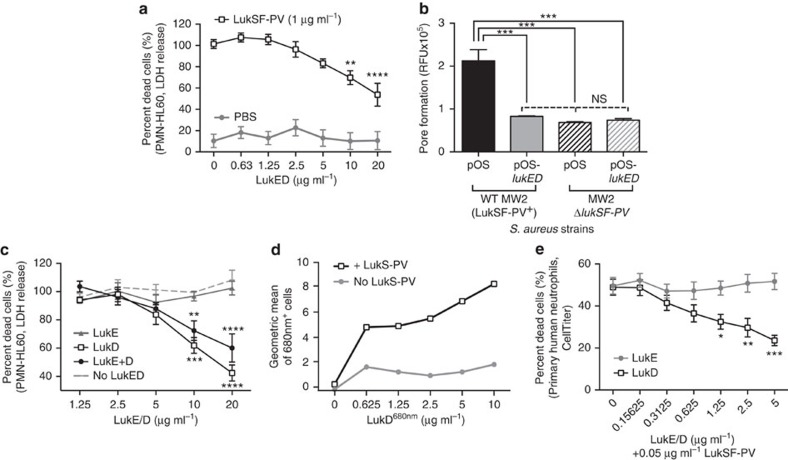
LukED inhibition of LukSF-PV-mediated toxicity on PMN-HL60 cells and primary human neutrophils. (**a**) PMN-HL60s were incubated with 1 μg ml^−1^ LukSF-PV or PBS in the presence of increasing concentrations of LukED. After 1 h incubation, cell lysis was monitored by measuring LDH release. (**b**) Culture supernatants from WT MW2 (WT LukSF-PV^+^) and an isogenic MW2 strain lacking *lukSF-PV* (Δ*lukSF-PV*) transformed with a plasmid overexpressing LukED (pOS-*lukED*), or a control plasmid (pOS) were incubated with PMN-HL60 cells at a final concentration of 10% (v/v). The formation of toxin-mediated pores in the plasma membrane of PMN-HL60s was monitored by ethidium bromide incorporation. (**c**) PMN-HL60 cells were first incubated with 1 μg ml^−1^ LukS-PV, any unbound LukS-PV washed away followed by addition of LukE and/or LukD, and finally, 1 μg ml^−1^ of LukF-PV. After 1 h incubation, cell lysis was monitored by measuring LDH release. (**d**) Binding of fluorescently labelled LukD^680 nm^ to PMN-HL60 cells, in the presence or absence of 0.25 μg ml^−1^ LukS-PV monitored by fluorescence-activated cell sorting. (**e**) Primary human neutrophils were incubated with 50 ng ml^−1^ LukSF-PV with increasing concentrations of LukE or LukD. After 1 h incubation, cell metabolism was monitored using the CellTiter reagent. Results represent the averages from three or more independent experiments±s.e.m. *P<0.05, **P<0.01, ***P<0.001 and ****P<0.0001 using one-way (**b**) or two-way analysis of variance (**a**,**c**,**e**). Results in part D are representative of three independent experiments.

The observation that PMN-HL60s are resistant to the hybrid leukotoxins, but not to LukSF-PV, prompted us to investigate if LukED and LukSF-PV could inhibit each other by forming inactive complexes. To this end, PMN-HL60 cells were incubated with LukSF-PV, in the presence or absence of increasing concentrations of LukED. As expected, in the absence of LukED, LukSF-PV resulted in complete lysis of PMN-HL60s. However, inclusion of LukED in 10–20 fold excess protected ∼50% of the cells (Fig. 1a).

Next, we determined if the inhibition observed with purified toxins could be recapitulated within the exoproteins in the milieu of *S. aureus* culture supernatants. During *in vitro* growth, *S. aureus* does not produce sufficient LukED to measure leukocidal activity^18^,^24^,^27^, while LukED is produced *in vivo* and contributes to the lethality of *S. aureus* bloodstream infection^9^,^10^,^24^. To overcome this limitation, a plasmid-based system was used to increase the expression of *lukED.* The *lukED*-expression plasmid (pOS-*lukED*), as well as the control plasmid (pOS), were transformed into the wildtype (WT) CA-MRSA USA400 strain MW2 (ref. 28) and an isogenic Δ*lukSF-PV* MW2 strain^29^. Ethidium bromide incorporation was used to monitor the formation of toxin-mediated pores in PMN-HL60s incubated with culture filtrates of the resulting strains (Fig. 1b). Heterologous production of LukED reduced the cytolytic activity of WT MW2 by ∼50% compared with the strain containing the control plasmid (WT/pOS versus WT/pOS-*lukED*). LukED reduced WT MW2 cytolytic activity to levels observed with the isogenic Δ*lukSF-PV* strain (WT/pOS-*lukED* versus Δ*lukSF-PV*/pOS). Moreover, production of LukED by the isogenic Δ*lukSF-PV* strain did not reduce the residual cytolytic activity of this strain, suggesting that LukED-mediated inhibition of the cytolytic activity by MW2 culture filtrates is specific to LukSF-PV.

### LukD inhibits LukSF-PV toxicity towards human neutrophils

Given the current model of leukotoxin assembly^6^,^12^, LukED-mediated inhibition of LukSF-PV lysis of PMN-HL60 cells could potentially occur by preventing LukS-PV from recruiting LukF-PV to form a pore. A sequential toxin subunit addition strategy was used to test this hypothesis. Cells were first incubated with LukS-PV, followed by removal of any unbound LukS-PV by washing. Cells were then incubated with LukE and/or LukD followed by extensive washing again, and final addition of LukF-PV. We observed that addition of LukED blocked LukSF-PV-mediated lysis (Fig. 1c). Moreover, comparison of the inhibitory activity of LukE and LukD revealed that LukD, but not LukE, is sufficient to inhibit LukSF-PV (Fig. 1c).

To further dissect the mechanism by which LukD blocked LukSF-PV-mediated killing of PMN-HL60 cells, a whole cell binding experiment was performed using a fluorescently labelled form of LukD (LukD^680 nm^). LukD^680 nm^ was found to exhibit minimal binding to PMN-HL60 cells. In contrast, upon binding to the surface of PMN-HL60s cells, LukS-PV was found to recruit LukD^680 nm^ to the plasma membrane of the cells (Fig. 1d). Altogether, these data support a model whereby LukD forms inactive complexes with LukSF-PV, thereby preventing LukS-PV from pairing with its cognate partner LukF-PV to form pores in the plasma membrane of PMN-HL60 cells.

Unlike PMN-HL60 cells, both standard leukotoxins (LukSF-PV and LukED) and their hybrid leukotoxins, can lyse primary human neutrophils^22^,^23^ (Supplementary Fig. 2D). Interestingly, when measuring cell metabolism as a readout for cytotoxicity^9^,^10^,^17^,^18^,^24^, instead of lactate dehydrogenase release, which is a measurement of cell lysis, LukSF-PV was found to be ∼10-fold more active than LukED and the hybrid leukotoxins (Table 1 and Supplementary Fig. 2D,E). Thus, we used this sensitive assay to test for potential inhibition of LukSF-PV by LukED. Primary human neutrophils were incubated with an LD_50_ of LukSF-PV in the presence of increasing concentrations of LukE or LukD, and cell viability monitored. These experiments revealed that LukSF-PV was inhibited by LukD, but not LukE (Fig. 1e), consistent with the findings with PMN-HL60 cells (Fig. 1c).

### LukSF-PV inhibits LukED toxicity towards murine leukocytes

**Figure 2:**
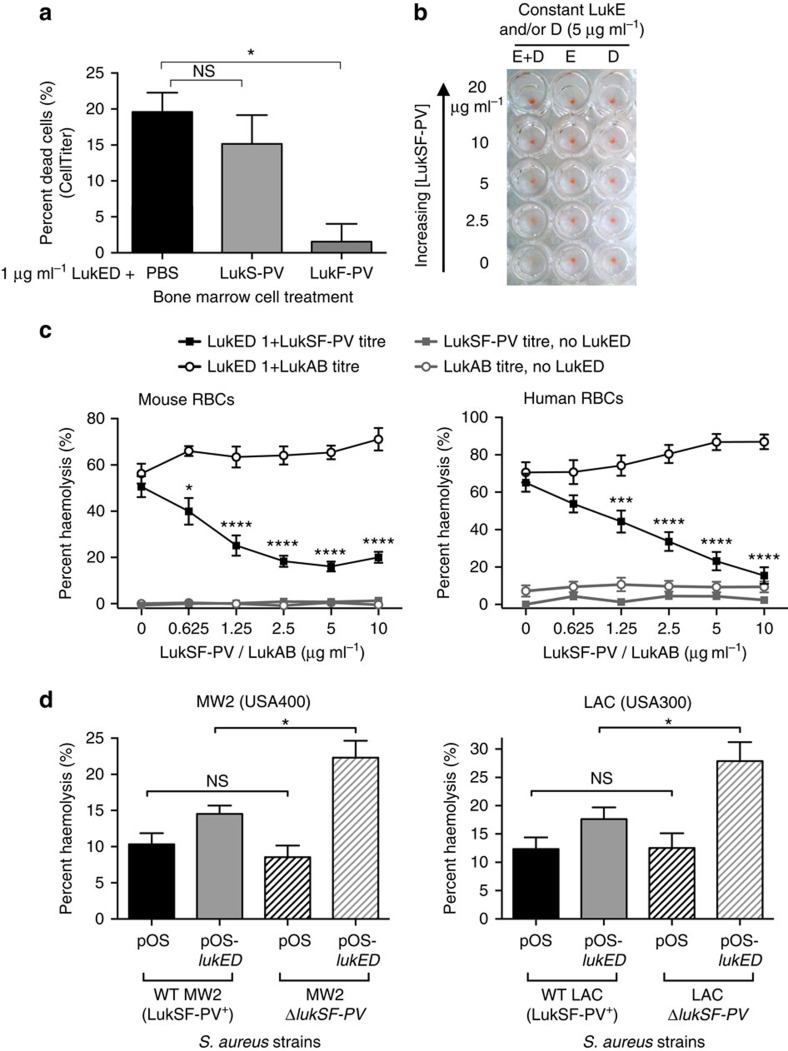
LukSF-PV inhibits LukED cytotoxic and haemolytic activity. (**a**) Primary murine bone marrow cells were incubated with LukED (1 μg ml^−1^) and either LukS-PV or LukF-PV (50 μg ml^−1^) for 1 h and cell metabolism measured using the CellTiter reagent. (**b**) Assay plate containing primary murine bone marrow cells in the presence or absence of indicated toxins, post centrifugation. Picture highlights red blood cell pellets in samples treated with LukED (E+D), LukE (E) or LukD (D) in the presence of increasing concentrations of LukSF-PV. (**c**) Primary murine (left panel) and primary human (right panel) RBCs were incubated with or without LukED in the presence of increasing concentrations of LukSF-PV or LukAB and haemolysis of the RBCs monitored. (**d**) Wildtype CA-MRSA strains USA400-MW2 and USA300-LAC (WT LukSF-PV^+^), and their isogenic Δ*lukSF-PV* counterparts were transformed with a plasmid overexpressing LukED (pOS-*lukED*), or a control plasmid (pOS), followed by incubation with human red blood cells at a MOI of 100. RBC lysis was measured at 405 nm after 1 h. Results represent the averages from three or more independent experiments±s.e.m. *P<0.05, **P<0.01, ***P<0.001 and ****P<0.0001 using one-way (**a**,**d**) or two-way analysis of variance (**c**).

To determine the potential contribution of LukED/LukSF-PV antagonism to *S. aureus* pathogenesis *in vivo*, we first tested if this phenomenon was observed using murine leukocytes. LukED is highly active against murine leukocytes, which has been shown to be critical for the lethality observed in mice infected systemically with *S. aureus*^9^,^10^,^24^. In contrast, LukSF-PV is unable to kill murine leukocytes (Table 2 and Supplementary Fig. 2F)^25^, a finding consistent with the specific tropism exhibited by this toxin towards the human C5aR receptor^11^. Thus, we evaluated the potential for LukSF-PV to inhibit LukED leukocidal activity. Primary murine leukocytes isolated from bone marrow, which consisted mostly of neutrophils, monocytes and macrophages, were incubated with LukED alone, or LukED with a 25-fold molar excess of LukS-PV, or LukF-PV. As expected, exposure to LukED resulted in the death of ∼20% murine leukocytes (Fig. 2a). LukED-mediated killing of these cells was found to be unaffected by the presence of excess LukS-PV, while LukF-PV protected the cells reducing cell death to <5% (Fig. 2a). Consistent with the data from PMN-HL60 cells and primary human neutrophils, it is the inert ‘F’ subunit of LukSF-PV that antagonizes the cytotoxic activity of LukED.

### LukSF-PV blocks LukED-mediated lysis of red blood cells

While performing the experiments with bone marrow-derived murine leukocytes we noticed that the cells treated with LukED in the presence of LukSF-PV had a notable residual red blood cell (RBC) pellet, which was absent from the cells treated with LukED alone (Fig. 2b). This observation suggested that LukSF-PV did not only inhibit LukED leukocidal activity, but it potentially blocked LukED haemolytic activity as well.

Among the leukotoxins, LukED and HlgACB toxins not only target leukocytes but also RBCs^6^. Both murine and human RBCs were highly susceptible to LukED (Table 2 and Supplementary Fig. 2G,H). In contrast, neither LukSF-PV, nor the hybrid combinations of LukE+LukF-PV or LukS-PV+LukD, exhibited any detectable haemolytic activity (Table 2 and Supplementary Fig. 2G,H).

To directly test if LukSF-PV inhibited LukED haemolytic activity, RBCs were incubated with LukED in the presence of increasing concentrations of LukSF-PV. In the absence of LukSF-PV, LukED resulted in lysis of ∼50% of murine RBCs and ∼70% of human RBCs (Fig. 2c). In contrast, when RBCs were incubated with LukED and LukSF-PV, ∼80–90% of the RBCs were protected from haemolysis. To ensure that this inhibition was specific for LukSF-PV, we also tested the haemolytic activity of LukED in the presence of LukAB, another non-haemolytic staphylococcal leukotoxin. We observed that in contrast to LukSF-PV, LukAB was unable to block LukED-mediated haemolysis (Fig. 2c).

To ensure that LukSF-PV-mediated inhibition of LukED haemolytic activity was not an effect only observed with purified toxins, we tested the haemolytic activity of isogenic WT and Δ*lukSF-PV* CA-MRSA representative strains, USA400 (MW2) and USA300 (LAC), transformed with a LukED-expression plasmid to increase the levels of LukED, or the corresponding empty vector control. In both WT CA-MRSA backgrounds, enhanced expression of LukED increased the haemolytic potential of those strains by ∼5%. In contrast, when LukED was produced in the Δ*lukSF-PV* backgrounds, the haemolytic potential of those strains was almost double that of the corresponding WT strains (Fig. 2d). These results indicate that the expression of LukSF-PV in both MW2 and LAC strains is sufficient to inhibit LukED haemolytic activity in the extracellular milieu of *S. aureus*.

### LukED lysis of human RBCs enhances *S. aureus* growth

**Figure 3:**
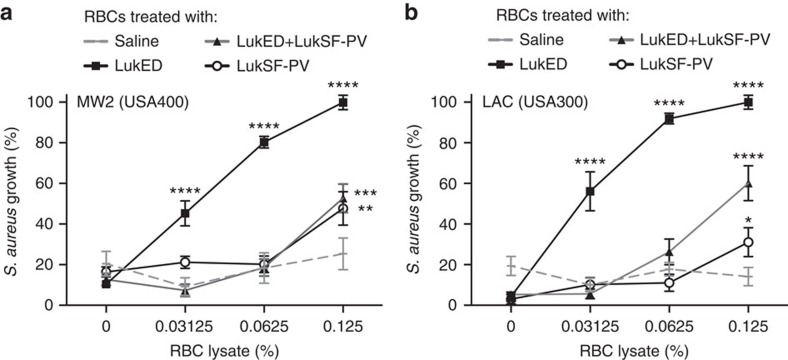
LukSF-PV inhibition of LukED-mediated haemolysis prevents the release of nutrients necessary for *S. aureus* growth. Iron-starved USA400-MW2 (**a**) and USA300-LAC (**b**) were subcultured into iron-restricted medium supplemented with increasing concentrations (v/v) of cell-free lysates of human RBCs treated with PBS, LukED (1 μg ml^−1^), LukSF-PV (10 μg ml^−1^), and a mixture of LukED (1 μg ml^−1^) and LukSF-PV (10 μg ml^−1^). Bacterial growth was monitored by measuring optical density, which was normalized to 100% of the maximal growth. Results represent the average from three or more independent replicates±s.e.m. **P<0.01, ***P<0.001 and ****P<0.0001 by two-way analysis of variance.

Iron is necessary for bacterial growth, but as there is little to no free iron in vertebrates. Bacterial pathogens have evolved sophisticated mechanisms to scavenge iron bound to host proteins^30^. *S. aureus* primarily scavenges iron from human haemoglobin thought to be released upon lysis of RBCs^31^,^32^,^33^,^34^,^35^. Thus, we reasoned that LukED-mediated lysis of RBCs could promote *S. aureus* growth, but not if haemolysis is inhibited by LukSF-PV. To directly test this possibility, cell-free supernatants were generated by treating human RBCs with LukED and/or LukSF-PV and used as nutrient sources. We observed impaired growth when two CA-MRSA strains, MW2 (Fig. 3a) and LAC (Fig. 3b), were inoculated into iron-starved medium supplemented with cell-free supernatants from RBCs treated with buffer or with LukSF-PV. In contrast, the strains were able to grow when inoculated into cell-free supernatants from the LukED treated human RBCs. Notably, bacterial growth was impaired in cell-free supernatants from RBCs treated with a mixture of LukED and LukSF-PV (Fig. 3a,b).

### Leukotoxin antagonism attenuates *S. aureus* infection in mice

**Figure 4:**
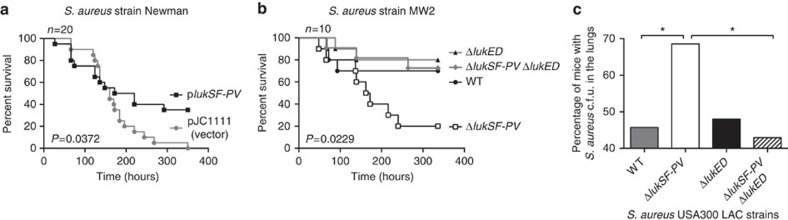
LukSF-PV inhibition of LukED attenuates *S. aureus in vivo*. (**a**) ‘Survival’ of mice infected with the isogenic Newman strains carrying the integrated empty plasmid (Newman::pJC1111; *n*=20) or the *lukSF-PV* containing plasmid (Newman::p*lukSF*-PV; *n*=20). (**b**) ‘Survival’ of mice infected with the isogenic USA400-MW2 strains (*n*=10). (**a**,**b**) Each mouse was infected intravenously with 5 × 10^7^ colony-forming units (c.f.u.) *S. aureus*. ‘Survival’ curves were compared using the log-rank (Mantel–Cox) test. (**c**) Pulmonary infections were established in mice by instilling 2 × 10^8^ c.f.u. of each isogenic USA300-LAC strains intranasally. Lungs were harvested 4 days post infection and plated for *S. aureus* counts. The data are represented as percentage of infected mice that harboured bacterial c.f.u. in the lungs 4 days post infection (*P*=0.0451). Statistical significance was determined using Fisher’s exact test.

Based on the observation that LukSF-PV blocks both LukED leukotoxic (Fig. 2a) and haemolytic activities (Fig. 2c) impacting *S. aureus* growth (Fig. 3a,b), we next explored if LukSF-PV also blocked LukED *in vivo* in a lethal LukED-mediated bloodstream infection model^9^,^10^,^24^. Of note, experiments evaluating the contribution of LukED to *S. aureus* pathogenesis have only been performed on strains that naturally lack *lukSF-PV*^9^,^10^,^24^. One such strain is Newman, a highly virulent MSSA strain commonly used in murine models of infection^9^,^10^,^24^,^36^,^37^,^38^,^39^,^40^,^41^,^42^,^43^,^44^. Thus, we engineered a Newman strain to express *lukSF-PV* from its endogenous promoter from a single copy in the chromosome at the SaPI1 attachment site^45^. Evaluation of the toxin profile by immunoblotting revealed that heterologous production of LukSF-PV did not affect the production of the other toxins in strain Newman (Supplementary Fig. 3A,C). Mice were then challenged with isogenic strains containing either an integrated empty vector or the vector containing the *lukSF-PV* locus and the development of acute disease monitored. While 100% of the mice infected with WT Newman succumbed to infection in the 2-week study, only 60% of the mice infected with the LukSF-PV producing strain succumbed to infection (Fig. 4a).

To further evaluate the effect of LukSF-PV to the pathogenesis of naturally LukSF-PV producing *S. aureus* strains, mice were also challenged with isogenic MW2 mutant strains (Fig. 4b and Supplementary Fig. 3B,D). In contrast to strain Newman, only 30% of the mice infected with WT MW2 succumbed to infection by the end of the 2-week study (Fig. 4b). Deletion of *lukSF-PV* (Δ*lukSF-PV*) had a profound effect on the pathogenesis of MW2, as 80% of the infected mice succumbed to infection. In contrast, deletion of *lukED* (Δ*lukED*) only exhibited a slight phenotype compared with the WT strain (20 versus 30%). To evaluate if the enhanced virulence by the Δ*lukSF-PV* strain was due to LukED, we examined the pathogenesis of an isogenic strain where the *lukED* locus was deleted in the Δ*lukSF-PV* background (Δ*lukSF-PV* Δ*lukED*). Deletion of *lukED* abolished the enhanced lethality exhibited by the Δ*lukSF-PV* strain (Fig. 4b), as the double mutant exhibited similar virulence as the Δ*lukED* strain.

Next, we sought to test if LukSF-PV inhibition of LukED altered the outcome of USA300 active infections. Unlike what we observed with the USA400-MW2 strains, we were unable to observe differences between WT and Δ*lukSF-PV* in mice infected with LAC in the bacteremia model (Supplementary Fig. 4A–C). However, upon intranasal infection^46^, although the overall bacterial burden between mice infected with the LAC strains did not differ, we noted that a significantly larger percentage of mice infected with the Δ*lukSF-PV* strain retained viable bacteria in their lungs (Fig. 4c and Supplementary Fig. 4D). These data suggest that deletion of *lukSF-PV*, thereby unrestricting LukED, promotes tissue colonization by USA300.

## Discussion

*S. aureus* uses a large arsenal of virulence factors to support its pathogenic lifestyle in humans^3^,^47^, including the production of up to five bi-component leukotoxins^6^. Leukotoxins have increasingly been recognized as important *S. aureus* virulence factors since their discovery more than a century ago^6^. However, research has predominantly focused on one toxin at a time with conclusions made with little consideration to the leukotoxins as a collective group. To our knowledge, we describe here for the first time the phenomenon we refer as ‘leukotoxin antagonism’, whereby leukotoxins can inhibit one another impacting the virulence of *S. aureus*.

**Figure 5:**
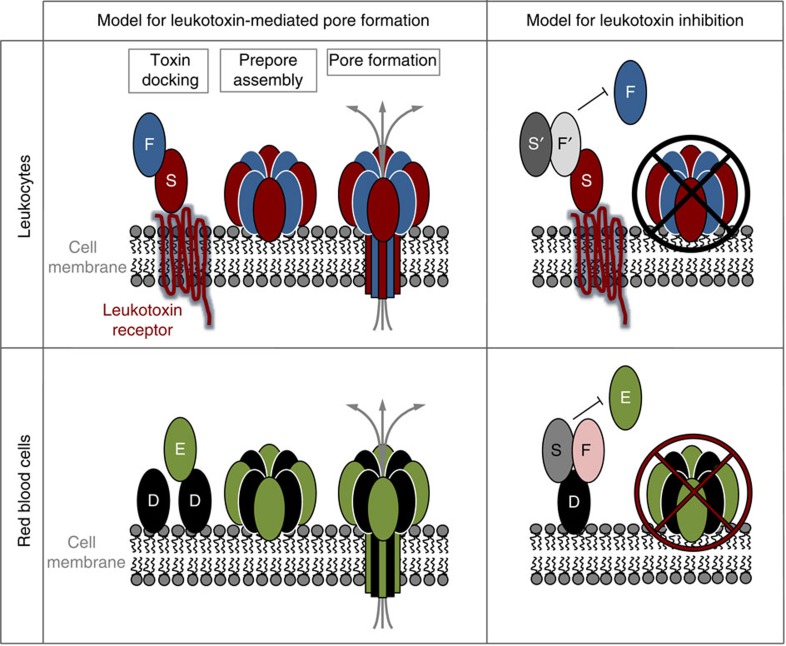
Models of leukotoxin antagonism. On leukocytes, an ‘F’ subunit from an ‘inert’ leukotoxin can associate with an ‘S’ subunit bound to that cell in a receptor dependent manner, giving rise to inactive hybrid complexes. Thus, the ‘S’ subunit is sequestered from its cognate ‘F’ partner, preventing the formation of pores in those cells. In RBCs, the formation of inactive hybrid complexes is different as the ‘F’ subunits interact with RBCs in what appears to be a receptor-independent manner, and is a prerequisite for haemolysis. Antagonism of LukED-mediated haemolysis in RBCs is observed by each LukS-PV or LukF-PV, although inhibition is enhanced in the presence of both LukS-PV and LukF-PV subunits.

Leukotoxin antagonism occurs in instances where a leukotoxin cannot target a certain cell type for lysis, but it can in turn interact with an active leukotoxin preventing the active toxin from exerting its cytotoxic effects. Interestingly, leukotoxin antagonism was also observed on human neutrophils, which are susceptible to all *S. aureus* leukotoxins, albeit to differing degrees. On leukocytes, we propose a model whereby an ‘inert’ ‘F’ subunit can form an inactive hybrid complex with a ‘S’ subunit bound to that cell, thereby preventing the ‘S’ subunit from pairing with its cognate ‘F’ partner to form pores in that cell (Fig. 5). Interaction of leukotoxins with RBCs, however, appear to be different, with nonspecific binding of ‘F’ subunits being a prerequisite for haemolysis (Supplementary Fig. 5A)^48^. Unlike leukotoxin inhibition of leukocyte lysis by an ‘inert’ ‘F’ subunit, LukS-PV and LukF-PV alone can equally inhibit LukED haemolysis, although inhibition is enhanced in the presence of both subunits (Fig. 5 and Supplementary Fig. 5B). Because the interaction of leukotoxins with RBCs is still not well understood, the mechanism of leukotoxin antagonism on these cells remains to be fully elucidated.

Leukotoxin antagonism was observed with both purified toxins and culture supernatants from isogenic *S. aureus* strains, on cell lines, primary leukocytes and primary RBCs. In addition, this phenomenon was demonstrated to influence the course of infection *in vivo* in two different models of murine infections with *S. aureus* strains representing different genotypes. A limitation of our studies is that while mice are an excellent model to study LukED, they are suboptimal for the study of LukSF-PV. Currently, there are no animal models that can fully recapitulate the susceptibility of human leukocytes to leukotoxins. Rabbits have been used to study LukSF-PV, however, deletions of *lukSF-PV* do not always manifest in differences in infection outcome^49^,^50^,^51^,^52^, despite the enhanced susceptibility of rabbit leukocytes to the cytolytic activity of LukSF-PV over that of murine cells^11^,^25^. Thus, additional research is needed to uncover the potential contribution of leukotoxin antagonism to *S. aureus* infection in humans.

Interestingly, leukotoxin antagonism manifested differently *in vivo* among different *S. aureus* strain backgrounds. While infection with isogenic mutants of strains MW2 and Newman resulted in overt differences in animal deaths, differences in pulmonary colonization was the only detectable phenotype for USA300-LAC strains. These strains reflect diverse *S. aureus* genotypes, namely, USA300, USA400 and CC8, suggesting that the phenomenon of leukotoxin antagonism occurs broadly across *S. aureus* strains. Importantly, our data also suggest that in addition to contributing to acute infection, leukotoxins could also be involved in the colonization process, which expands the function of these virulence factors.

As an excess of the inhibiting toxin subunit(s) is necessary for antagonism to occur, and *S. aureus* appears to express higher levels of LukSF-PV than LukED under many conditions tested, we postulate that LukSF-PV inhibition of LukED may be more prevalent in the clinical setting. We envision that the occurrence of leukotoxin inhibition *in vivo* during active infection in the murine models could extend to *S. aureus* infection of humans given that LukSF-PV also inhibits LukED haemolysis of human RBCs, and that a majority of CA-MRSA strains express Panton–Valentine leukocidin. Based on our findings, we propose a model where LukSF-PV blocks LukED haemolytic activity *in vivo*, which in turn slows *S. aureus* replication by limiting the bioavailability of nutrients required for bacterial growth. Slowing bacterial growth may help establish long-term infections and possibly assist *S. aureus* subversion of host immunity. Importantly, several independent groups have reported that deletion of *lukSF-PV* in CA-MRSA strains, including the epidemic strain in the United States, USA300, increases the virulence of the tested strain in murine models of pneumonia, systemic and skin abscess infections^29^,^53^,^54^,^55^. These phenotypes were initially attributed to the proinflammatory role of LukSF-PV in activating innate immunity^54^,^55^. However, the findings described here suggest that leukotoxin antagonism is more likely responsible for these phenotypes. It should be noted that the potential clinical relevance of LukED antagonism of LukSF-PV certainly cannot be ruled out at this point either, especially given the major role neutrophils play in immune defence against *S. aureus*^47^.

We are in the early stages of uncovering why *S. aureus* produces numerous related leukotoxins and how they contribute to disease. Leukotoxins exhibit unique attributes and as such, they likely have distinct roles during infection^6^. It remains to be elucidated if the large numbers of toxins produced by *S. aureus* exist solely to increase the amount of cellular targets required for efficient immune cell lysis by the bacterium. Alternatively, the interplay between these leukotoxins could also modulate *S. aureus* pathogenesis, beyond merely promoting cell lysis. We postulate that while the cytotoxic capabilities of leukotoxins likely have a role in establishing infections, the ability of leukotoxins to inhibit each other within certain environments could be beneficial for *S. aureus* as it has the potential to diminish overt tissue damage, inflammation and ultimately death of the infected host. As such, leukotoxin antagonism could impact the development of anti-toxin strategies by adding a new layer of complexity previously unknown. Our study stresses the importance of deciphering how bi-component leukotoxins work *in vivo* as a collective group of virulence factors, as this information is required for the development of more effective anti-toxin treatments to combat *S. aureus* infections.

## Methods

### Murine *S. aureus* septicaemia infection

All animal infections were performed according to protocols approved by the NYU School of Medicine Institutional Animal Care and Use Committee. Female ND4 Swiss Webster mice (Harlan Laboratories), at ∼6 weeks of age, were used for intravenous infections, where *S. aureus* strains were injected retro-orbitally at 5 × 10^7^ colony-forming unit (c.f.u.). Time taken for each animal to develop acute infection was recorded as we have carried out before^24^. Every effort was taken to humanely sacrifice moribund mice before death occurs, hence infection outcomes are reported as ‘survival’ curves. Female BALB/c mice (Jackson Laboratories), at ∼7 weeks of age, were used for intranasal infections. Each mouse was instilled with a 30-μl suspension containing 2–4 × 10^8^ c.f.u. *S. aureus* into the right nostril. Lungs were collected 4 days post infection and plated for *S. aureus* counts.

### Cell lines

HL60 cells (ATCC CCL-240), a human promyelocytic cell line, were cultured in Roswell Park Memorial Institute 1640 medium (RPMI, Cellgro) supplemented with 10% heat inactivated fetal bovine serum (FBS) and penicillin-streptomycin at 37 °C with 5% CO_2_. Differentiation of HL60 cells into neutrophil-like cells (PMN-HL60s) was achieved by supplementing 1.5% (v/v) dimethylsulfoxide in the culture medium for 3 days, while differentiation into macrophage-like cells was carried out by incubating cells with 16-nM phorbol 12-myristate 13-acetate for 4 days. All assays were conducted with cells at 10^6^ ml^−1^, with the exception of flow cytometry experiments where cells were reduced to a density of 5 × 10^5^ ml^−1^.

### Leukotoxin purification, fluorescent labelling and binding assay

His-tagged fusions of unlabelled subunits of the leukotoxins LukSF-PV, LukED and LukAB were purified from *E. coli* lysates or *S. aureus* supernatants as described elsewhere^17^,^24^.

The labelling of the LukD subunit with a 680-nm fluorescent tag (LukD^680 nm^) was carried out using a Thermo Scientific Dylight Amine Reactive Dye according to manufacturer’s instructions. To determine binding of LukD^680 nm^ to PMN-HL60 cells, increasing concentrations of LukD^680 nm^ were incubated with 5 × 10^5^ ml^−1^ PMN-HL60 cells on ice for 10 min. Cells were washed once with RPMI+10% FBS, followed by data acquisition on an LSRII flow cytometer (BD Biosciences) using the FACSDiva software. Data were analysed using the FlowJo software (Treestar).

### Bacterial strains and media

The *S. aureus* CA-MRSA USA400 strain MW2, in addition to the MW2 isogenic Δ*lukSF-PV::erm* (Δ*lukSF-PV*) knockout strain have previously been described^28^,^29^. A *lukED* mutant was generated in MW2 using allelic replacement, giving rise to MW2 Δ*lukED::kan* (Δ*lukED*)^24^. The double MW2 Δ*lukED* Δ*lukSF-PV* mutant was generated by transducing the MW2 Δ*lukSF-PV* strain with phage lysate from the MW2 Δ*lukED::kan* strain, followed by selection for the Δ*lukED::kan* locus on media containing kanamycin. A comprehensive set of isogenic mutants (Δ*lukED::kan*, Δ*lukSF-PV::spectinomycin* (spec) and Δ*lukED::kan* Δ*lukSF-PV::spec*) were similarly generated in an erythromycin-sensitive derivative of the USA300 clinical strain LAC^56^. WT and Δ*lukSF-PV* strains of MW2 and LAC were also transformed with the plasmids pOS1-P_*lukAB*_-*sslukA*-6His (referred to in the text as pOS) and pOS1-P_*lukAB*_-*sslukA*-6His-*lukED* (referred to in the text as pOS-*lukED*)^17^, followed by selection on chloramphenicol.

Bacterial growth media used in this study include Luria-Bertani and tryptic soy broth/agar (TSB/A), unless otherwise indicated. All strains were grown in broth culture overnight, with appropriate antibiotics for strains harbouring plasmids. After overnight culture, strains were subcultured 1:100 into fresh media without antibiotics and grown with shaking for 5 h at 37 °C. Filter sterilized supernatants from *S. aureus* cultures were used in cytotoxicity assays, in addition to precipitation with 10% trichloroacetic acid for confirmation of cytotoxin profiles by western blot. For mouse infections, *S. aureus* strains were grown in TSB for 3 h, followed by washing with PBS as previously described.

To generate a LukSF-PV-expressing *S. aureus* strain Newman^36^, the *lukSF-PV* locus with its endogenous promoter (P_*lukSF-PV*_-*lukSF-PV*) were introduced into the SaPI1 attachment site in the chromosome^45^. Briefly, P_*lukSF-PV*_-*lukSF-PV* was PCR amplified from the strain MW2 as previously described^54^. Following restriction digests with BamHI and PstI, the P_*lukSF-PV*_-*lukSF-PV* fragment was cloned into the integration vector pJC1111 similarly digested with BamHI and PstI. The empty pJC1111 vector was used as a control. Protocol for the integration of pJC1111 into the *S. aureus* genome has been outlined elsewhere^24^,^27^,^45^.

### Cytotoxicity and pore formation assays

Cell lysis was measured by the release of the cytosolic enzyme lactate dehydrogenase according to manufacturer’s protocol (CytoTox-ONE Homogenous Membrane Integrity Assay, Promega). Pore formation was assessed by cellular ethidium bromide incorporation by including ethidium bromide in the assay medium at 5 μg ml^−1^. Metabolism by viable cells was measured using the colorimetric reagent CellTiter 96 AQueous One Solution Cell Proliferation Assay (Promega).

For these assays, purified toxins or filter sterilized *S. aureus* supernatants were incubated with cells for 1 h at 37 °C with 5% CO_2_. In experiments measuring LukED inhibition of LukSF-PV toxicity on PMN-HL60 cells by sequential leukotoxin subunit addition, there was a 30-min incubation at 37 °C with 5% CO_2_ following the addition of each subunit. The cells were then washed with PBS before addition of the next subunit.

### Isolation of mouse bone marrow cells

Upon euthanasia, bone marrow cells were flushed from bilateral tibias and femurs with PBS. For intoxication with *S. aureus* leukotoxins, cell densities were adjusted to 10^6^ per ml with RPMI containing 10% FBS.

### Studies using human and mouse RBCs

RBCs from Swiss Webster mice were collected in Alsever’s solution by submandibular bleeding. Human blood preserved in heparin or a citrate phosphate double dextrose solution was purchased from the New York Blood Center, where written informed consent was obtained from all participants involved in the study.

RBCs were washed three times with 0.9% sodium chloride USP (saline) followed by centrifugation at 600*g* for 10 min. After washing, RBCs were serially diluted in saline, and optical density was read at 405 nm (OD_405_) to determine the relative amounts of haemoglobin in each dilution. For subsequent assays, RBC dilutions with OD_405_ of 0.15 were used.

For measurement of RBC lysis, 80 μl of RBC were aliquoted per well of a v-bottom 96-well plate and toxin (or saline) added to a final reaction volume of 100 μl. In assays where potential LukSF-PV inhibition of toxin haemolysis was measured, RBCs were preincubated with LukSF-PV or LukAB at room temperature for 10 min before addition of the haemolytic leukotoxin. The plates were incubated at 37 °C with 5% CO_2_ for 30 min, followed by immediate centrifugation at 1,500 r.p.m. for 5 min. A measure of 50 μl of supernatant was transferred to a flat bottom microtiter plate and optical density read at 405 nm.

USA300 and USA400 strains used for infection of human RBCs were grown overnight in TSB containing chloramphenicol for plasmid maintenance. The following day, the strains were subcultured 1:100 into RPMI+5% casamino acids for 5 h. The cultures were centrifuged and adjusted to OD_600_ of 1 with saline. The bacterial suspensions and normalized RBCs were mixed 1:1 in v-bottom plates for an MOI of 100, synchronized by centrifugation at 1,500  r.p.m. for 5 min, followed by incubation at 37 °C for 1 h. Supernatants from the infections were transferred to flat bottom plates and read at 405 nm for measurement of RBC lysis.

### Haemoglobin-dependent *S. aureus* growth

*S. aureus* colonies were restricted of iron by overnight growth in 2 ml RPMI supplemented with 1 μM of the iron chelator ethylenediamine-di(o-hydroxyphenylacetic acid) (LGC Standards GmbH)^35^. The overnight cultures were normalized to OD_600_ of 0.5. Bacteria were then inoculated (1:200 final dilution) into cell-free supernatants from human RBCs generated by incubation with saline control, 1 μg ml^−1^ LukED, 1 μg ml^−1^ LukED and 10 μg ml^−1^ LukSF-PV and 10 μg ml^−1^ LukSF-PV, for 30 min at 37 °C. Iron restricted *S. aureus* were incubated with human RBC lysates in 96-well round bottom plates with shaking at 37 °C, and growth of *S. aureus* was measured after 24 h by optical density at 600 nm.

### Statistical analyses

Statistical analyses were performed with Prism (GraphPad Software). Statistics for ‘survival’ curves were calculated using a log-rank (Mantel–Cox) test. Statistical significance was calculated using Fisher’s exact test and analysis of variance with Dunnett’s multiple comparisons post test where appropriate.

## Additional information

**How to cite this article:** Yoong, P. & Torres, V. J. Counter inhibition between leukotoxins attenuates *Staphylococcus aureus* virulence. *Nat. Commun.* 6:8125 doi: 10.1038/ncomms9125 (2015).

## Supplementary Material

Supplementary Information Supplementary Figures 1-5

## Figures and Tables

**Table 1:** Susceptibility of the human promyelocytic cell line HL60 (undifferentiated and differentiated) and primary human neutrophils to LukSF-PV, LukED and their hybrid leukotoxins.

	**Standard toxins**		**Hybrid toxins**	
**Cells**	**LukSF-PV**	**LukED**	**LukS-PV+ LukD**	**LukE+ LukF-PV**
Undifferentiated HL60 cells	−*	+†	−	+
Macrophage-like HL60 cells	+++‡	−	++§	−
PMN-like HL60 cells	++++||	−	−	−
Primary human neutrophils	++++	+++	+++	+++

LukED, leukocidin ED; LukSF-PV, Panton–Valentine leukocidin; PMN, poly-morphonuclear neutrophils.

^*^− No detectable lytic activity

^†^+ LD_50_ >20 μg ml^−1^

^‡^+++ LD_50_ 1–5 μg ml^−1^

^§^++ LD_50_ 5–20 μg ml^−1^

^||^++++ LD_50_ <1 μg ml^−1^

**Table 2:** Susceptibility of murine bone marrow cells, and murine and human red blood cells to LukSF-PV, LukED and their hybrid leukotoxins.

	**Standard toxins**		**Hybrid toxins**	
**Cells**	**LukSF-PV**	**LukED**	**LukS-PV+LukD**	**LukE+LukF-PV**
Mouse BM	**−** *	**++** †	**−**	**−**
Mouse RBCs	**−**	**++++** ‡	**−**	**−**
Human RBCs	**−**	**++++**	**−**	**−**

BM, bone marrow; LukED, leukocidin ED; LukSF-PV, Panton–Valentine leukocidin; RBC, red blood cells.

^*^− No detectable lytic activity

^†^++ LD_50_ >5 μg ml^−1^

^‡^++++ LD_50_ <1 μg ml^−1^
