# Supplementary material for: Counter inhibition between leukotoxins attenuates Staphylococcus aureus virulence
Source: Nat Commun. 2015 Sep 2;6:8125. doi: 10.1038/ncomms9125 (PMC4562310; doi:10.1038/ncomms9125)
Supplement: Supplementary Information — Supplementary Figures 1-5 [file ncomms9125-s1.pdf]

Alignment of 'S' subunits

LukS-PV & LukE → 71% identity

```
LukS-PV    NNIENIGDGAEVVKRTEDTSSDKWGVTQNIQFDFVKDKKYNKDALILKMQ  50
LukE       TNIENIGDGAEVIKRTEDVSSKKWGVTQNVQFDFVKDKKYNKDALIVKMQ  50

LukS-PV    GFINSKTTYYNYKNT--DHIKAMRWPFQYNIGLKTNDPNVDLINYLPKNK  98
LukE       GFINSRTSFSDVKGSGYELTKRMIWPFQYNIGLTTKDPNVSLINYLPKNK  100

LukS-PV    IDSVNVSQTLGYNIGGNFNSGPSTGGNGSFNYSKTISYNQQNYISEVERQ  148
LukE       IETTDVGQTLGYNIGGNFQSAPSIGGNGSFNYSKTISYTQKSYVSEVDKQ  150

LukS-PV    NSKSVQWGIKANSFITSLGKMSGHDPNLFVG-YKPYSQNPRDYFVPDNEL  197
LukE       NSKSVKWGVKANEFVTPDGKKSAHDRYLFVQSPNGPTGSAREYFAPDNQL  200

LukS-PV    PPLVHSGFNPSFIATVSHEKGSGDTSEFEITYGRNMDVTHATRRTTHYGN  247
LukE       PPLVQSGFNPSFITTLSHEKGSSDTSEFEISYGRNLDITYAT----LFPR  246

LukS-PV    SYLEGSRIHNAFVNRNYTVKYEVNWKTHEIKVKGHN  283
LukE       TGIYAERKHNAFVNRNFVVRYEVNWKTHEIKVKGHN  282
```

Alignment of 'F' subunits

LukF-PV & LukD → 82% identity

```
LukF-PV    AQHITPVSEKKVDDKITLYKTTATSDSDKLKISQILTFNFIKDKSYDKDT  50
LukD       AQHITPVSEKKVDDKITLYKTTATSDNDKLNISQILTFNFIKDKSYDKDT  50

LukF-PV    LILKAAGNIYSGYTKPNPKDTISSQFYWGSKYNISINSDSNDSVNVVDYA  100
LukD       LVLKAAGNINSGYKKPNPKDYNYSQFYWGGKYNVSVSSESNDAVNVVDYA  100

LukF-PV    PKNQNEEFQVQQTVGYSYGGDINISNGLSGGGNGSKSFSETINYKQESYR  150
LukD       PKNQNEEFQVQQTLGYSYGGDINISNGLSGGLNGSKSFSETINYKQESYR  150

LukF-PV    TSLDKRTNFKKIGWDVEAHKIMNNGWGPYGRDSYHSTYGNEMFLGSRQSN  200
LukD       TTIDRKTNHKSIGWGVEAHKIMNNGWGPYGRDSYDPTYGNELFLGGRQSS  200

LukF-PV    LNAGQNFLEYHKMPVLSRGNFNPEFIGVLSRKQNAAKKSKITVTYQREMD  250
LukD       SNAGQNFLPTHQMPLLARGNFNPEFISVLSHKQNDTKKSKIKVTYQREMD  250

LukF-PV    RYTNFWNQLHWIGNNYKDENRATHTSIYEVDWENHTVKLIDTQSKEKNPM  300
LukD       RYTNQWNRLHWVGNNYKNQNTVTFTSTYEVDWQNHTVKLIGTDSKETNPG  300

LukF-PV    S 301
LukD       V 301
```

Supplementary Figure 1

Amino acid sequence similarity between LukSF-PV and LukED.

Amino acid sequences o the mature secreted 'S' and 'F' subunits of LukSF-PV and LukED were aligned using the Clustal W2 program. Black boxes indicate fully conserved residues, gray boxes indicate conservation between amino acid residues of strongly similar properties, while gray letters denote residues of weakly similar properties.

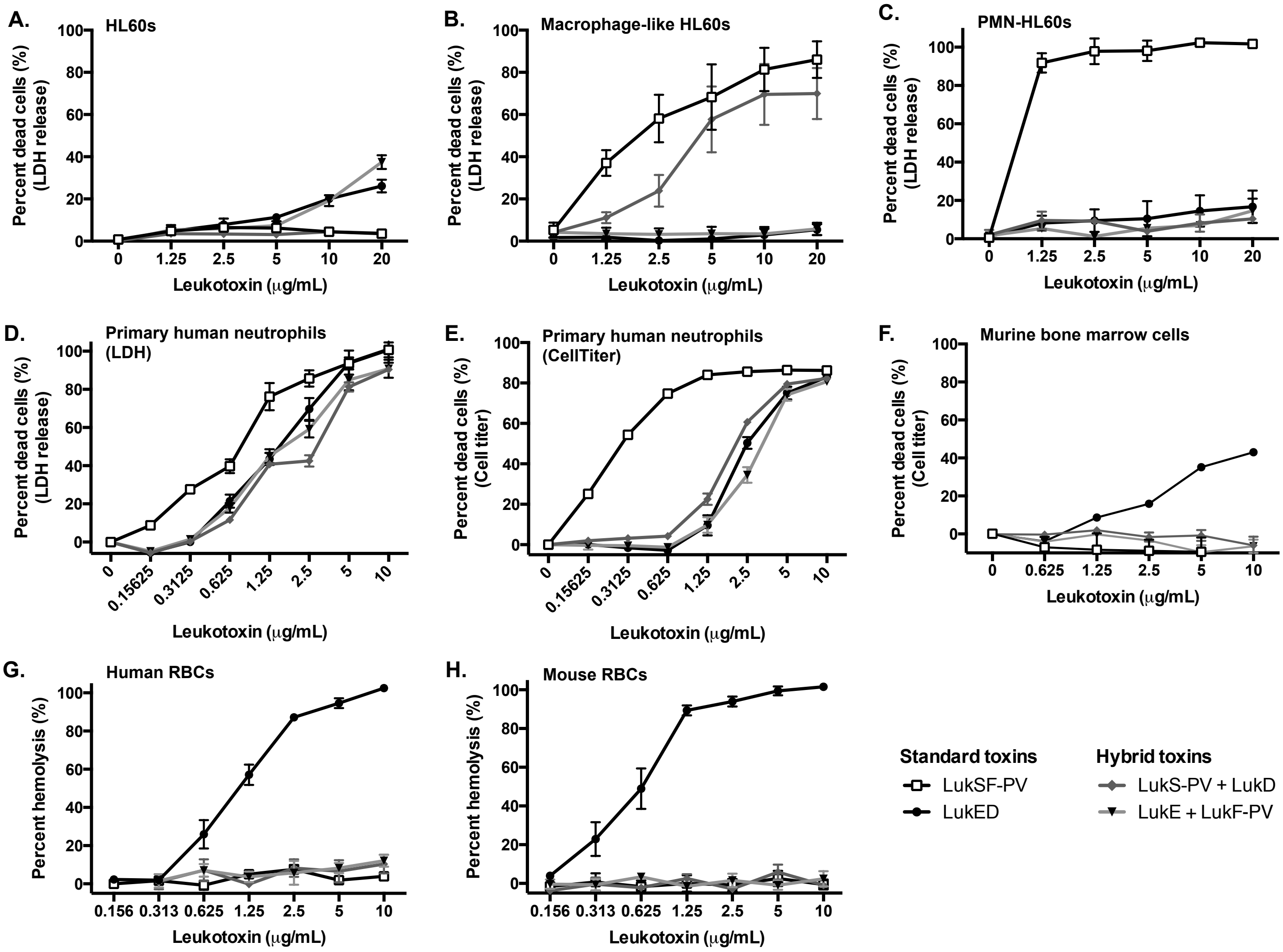

Supplementary Figure 2

Susceptibility of human and murine cells to LukSF-PV, LukED and their hybrid leukotoxins.

Cytotoxic activities of native and hybrid LukED and LukSF-PV toxins on human HL60 cells: undifferentiated (A), differentiated into macrophage-like cells (B), differentiated into neutrophil-like cells (C), as well as primary human neutrophils (D) were monitored by measuring LDH release after one hour. Cytotoxic activities of the leukotoxins were also measured on primary human neutrophils (E), and murine bone marrow cells (F) using the cell metabolism indicator CellTiter. Hemolytic activity was measured by hemoglobin release from human (G) and murine (H) red blood cells. Results represent the averages from two or more independent experiments ± SEM.

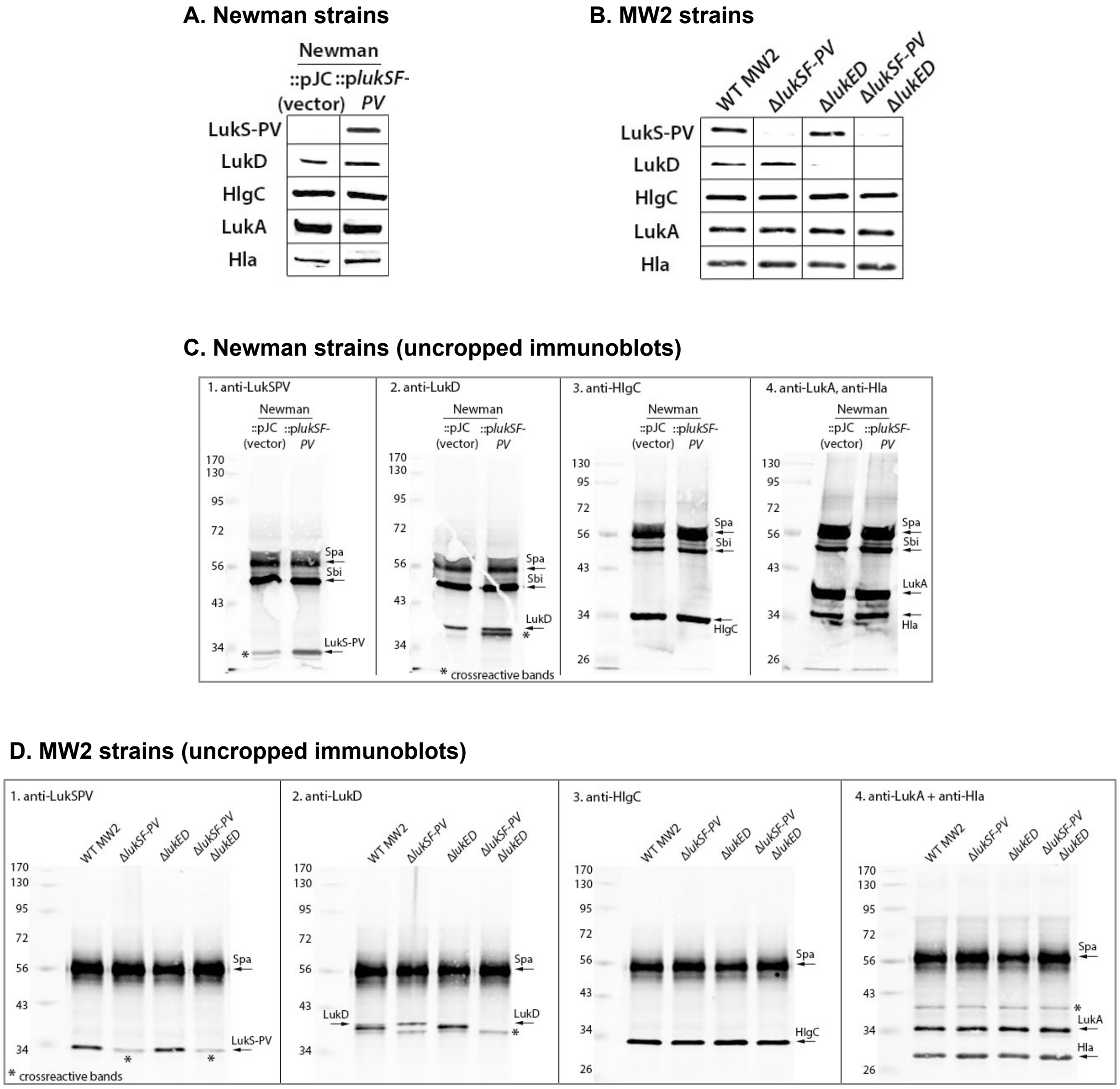

Supplementary Figure 3

Toxin production by the Newman and MW2 strains used in animal infections.

Toxin profiles of culture filtrates isolated from isogenic Newman (A,C), and MW2 (B,D) strains, as determined by immunoblotting with toxin-specific sera. Panels A and B represent cropped toxin bands, while panels C and D represent uncropped immunoblots. Spa and Sbi are antibody-binding proteins that are prevalent in *S. aureus* strains. Note that Sbi expression is elevated in strain Newman compared with other *S. aureus* strains.

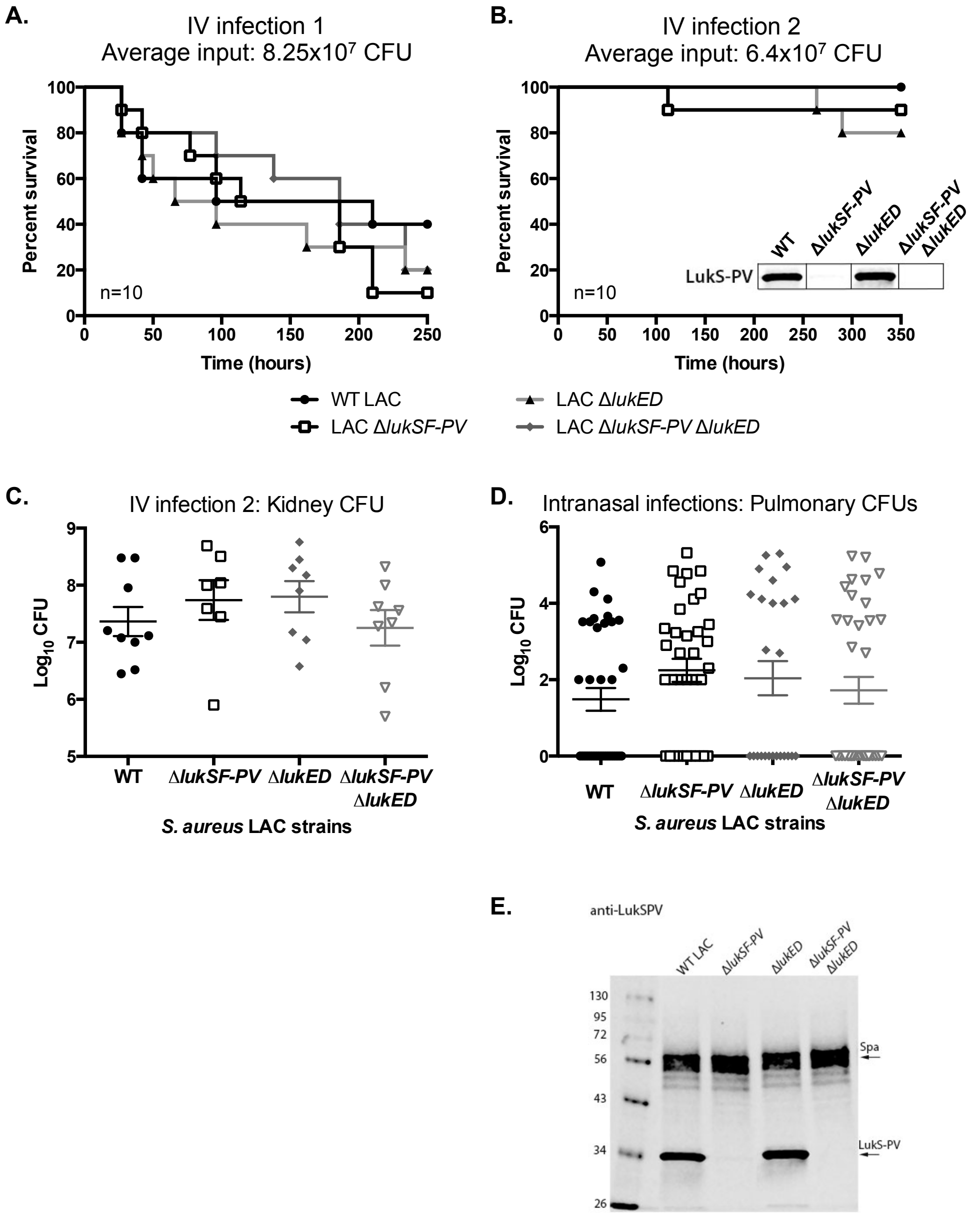

Supplementary Figure 4

Effect of LukSF-PV expression on LukED pathogenesis of USA300 strain LAC in murine models of infections.

A, B. “Survival” curves of intravenous infection with isogenic USA300 LAC strains. Inset: Immunoblots showing toxin production by LAC strains. C. Bacterial counts from kidneys of mice 15 days post IV infection (from infection 2 in panel B). D. Bacterial counts from lungs of mice 4 days after intranasal infection with $2x10^8$ CFU (see also Fig 4C). The bacterial load between strains in C and D were analyzed by one-way ANOVA, and found not to be statistically significant. E. Uncropped immunoblot of LukSF-PV expression from isogenic LAC strains.

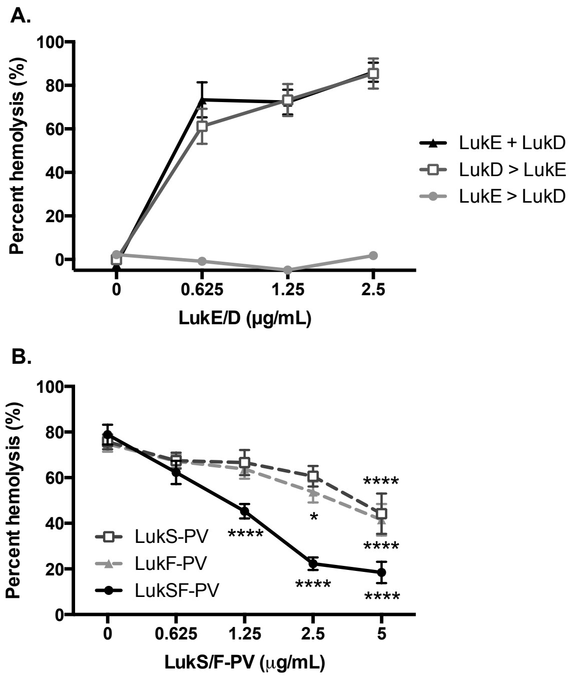

Supplementary Figure 5

Mechanisms of leukotoxin recruitment (A) and antagonism (B) on RBCs differ from those on leukocytes.

A. Leukotoxin mediated lysis of RBCs. Human RBCs were incubated with increasing concentrations of (i) LukE, washed and then LukD (LukE > LukD), (ii) LukD, washed and then LukE (LukD > LukE), or with (iii) LukE+LukD together (LukED), and hemolysis of the RBCs monitored by hemoglobin release.

B. Human RBCs were incubated with LukED (1μg/mL) in the presence of increasing concentrations of LukSF-PV, LukS-PV, or LukF-PV and hemolysis of the RBCs monitored.

Results represent the average from three or more independent experiments ± SEM. * $P < 0.05$, *** $P < 0.001$ and **** $P < 0.0001$ by two-way ANOVA.
